# Friends Turned Foes: Angiogenic Growth Factors beyond Angiogenesis

**DOI:** 10.3390/biom7040074

**Published:** 2017-10-02

**Authors:** Pratiek N. Matkar, Ramya Ariyagunarajah, Howard Leong-Poi, Krishna K. Singh

**Affiliations:** 1 Division of Cardiology, Keenan Research Centre for Biomedical Science, St. Michael’s Hospital, Toronto, ON M5B 1W8, Canada; matkarp@smh.ca (P.N.M.); leong-poih@smh.ca (H.L.-P.); 2 Institute of Medical Science, University of Toronto, Toronto, ON M5S 1A8, Canada; 3 Windsor University School of Medicine, Cayon, Saint Kitts and Nevis; ramya.ariyam@gmail.com; 4 Division of Vascular Surgery, Keenan Research Centre for Biomedical Science, St. Michael’s Hospital, Toronto, ON M5B 1W8, Canada; 5 Department of Pharmacology and Toxicology, University of Toronto, Toronto, ON M5S 1A8, Canada; 6 Department of Surgery, University of Toronto, Toronto, ON M5S 1A8, Canada

**Keywords:** angiogenesis, growth factors, pathologies, therapeutic targets

## Abstract

Angiogenesis, the formation of new blood vessels from pre-existing ones is a biological process that ensures an adequate blood flow is maintained to provide the cells with a sufficient supply of nutrients and oxygen within the body. Numerous soluble growth factors and inhibitors, cytokines, proteases as well as extracellular matrix proteins and adhesion molecules stringently regulate the multi-factorial process of angiogenesis. The properties and interactions of key angiogenic molecules such as vascular endothelial growth factors (VEGFs), fibroblast growth factors (FGFs) and angiopoietins have been investigated in great detail with respect to their molecular impact on angiogenesis. Since the discovery of angiogenic growth factors, much research has been focused on their biological actions and their potential use as therapeutic targets for angiogenic or anti-angiogenic strategies in a context-dependent manner depending on the pathologies. It is generally accepted that these factors play an indispensable role in angiogenesis. However, it is becoming increasingly evident that this is not their only role and it is likely that the angiogenic factors have important functions in a wider range of biological and pathological processes. The additional roles played by these molecules in numerous pathologies and biological processes beyond angiogenesis are discussed in this review.

## 1. Introduction

Angiogenesis is described as the development of new blood vessels from pre-existing ones. Under normal physiological conditions, the tightly regulated angiogenic process is essential for embryonic growth, wound healing and the female reproductive system [1,2]. Primarily, angiogenic growth factors such as vascular endothelial growth factors (VEGFs) and fibroblast growth factors (FGFs) induce the secretion of endothelial proteinases and plasminogen activators that cause the breakdown of the vessel basement membrane, allowing the cells to intrude the adjoining matrix [2]. Subsequently, the endothelial cells migrate, multiply and ultimately differentiate to give rise to a new, lumen-comprising vessel. Thereafter, the endothelial cells establish a new basement membrane and release additional factors such as platelet-derived growth factor (PDGF), which draws the supporting pericytes to interact externally with the endothelial cells in order to stabilize the newly formed vessels [2].

Angiogenic sprouting is coordinated by a gentle balance between several pro- and anti-angiogenic factors, such as VEGFs, FGFs, angiopoietins (Ang1–4), PDGF, transforming growth factor beta (TGFβ), tumor necrosis factor alpha (TNFα), integrins, adhesion molecules and matrix degrading enzymes [3]. Among the inducers of angiogenesis, the VEGFs, FGFs, and angiopoietins are well-studied and characterized, and probably the most essential angiogenic molecules. Therefore, the current review will focus on these three families of angiogenic growth factors.

**Figure 1:**
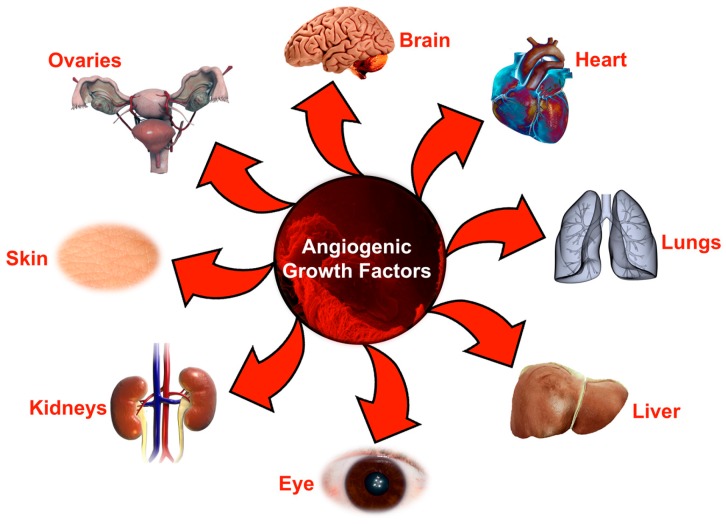
Organs affected by angiogenic growth factors during various pathological conditions. Schematic diagram representing the most important organs in the body that are usually affected by impaired or excessive angiogenic signaling during initiation or progression of various pathologies.

Several pathological conditions, such as rheumatoid arthritis, diabetes, chronic kidney disease, cutaneous complications, neurodegenerative disorders, cancer and age-related macular degeneration (Figure 1) are characterized by excessive angiogenesis, where vessels grow in an unrestrained and deranged manner. Moreover, several pathological conditions also exist where these growth factors play diverse roles beyond angiogenesis such as modulation of the immune system, lipid metabolism, glucose metabolism, cellular differentiation and podocyte damage. In this review, we discuss several studies that underline new insights into how these angiogenic growth factor signaling pathways go awry in various pathological conditions. Finally, our review will shed light upon many exciting utilities of these growth factors as diagnostic agents as well as therapeutic targets.

## 2. Vascular Endothelial Growth Factor

Till date, there have been seven members identified in the VEGF family of cytokines: VEGF-A, VEGF-B, VEGF-C, VEGF-D, VEGF-E, VEGF-F, and Placental growth factor (PIGF) [4]. VEGF-A (commonly referred to as VEGF) exists as six mRNA splice variants—isoforms 121, 145, 165, 183, 189, and 206, depending upon the number of amino acids present [5]. All VEGF family members are characterized by the presence of a common VEGF homology domain and further consist of various isoforms with diverse functions in the human body. The VEGFs generally bind to three tyrosine kinase receptors: the fms-like tyrosine kinase (Flt-1/VEGF receptor-1; VEGFR-1), the fetal liver kinase (Flk-1/VEGFR-2/KDR), and Flt-4 (VEGFR-3) [4]. VEGFR-2 has been identified as the primary receptor for mediating mitogenesis and regulating vascular permeability [6,7]. Although the function of VEGFR-2 in endothelial cells has been investigated extensively, VEGFR-1 remains a relatively less studied receptor. Interestingly, in spite of having the greatest affinity for VEGF-A, VEGFR-1 possesses a relatively lower intrinsic kinase activity when compared to VEGFR-2, resulting in its inability to mediate mitogenesis [8]. However, VEGFR-1 plays important roles in endothelial cell division during the initial phases of vasculature development. Unlike VEGFR-1/2, VEGFR-3 is able to bind only VEGF-C and VEGF-D [9,10,11]. VEGFR-3 expression is primarily limited to lymphatic endothelial cells where it mediates a plethora of functions like cell migration, survival, and differentiation. VEGF-C binds to neuropilin-2 (NRP-2) and significantly facilitates lymphatic growth in cooperation with VEGFR-3 [9]. Additionally, a VEGF-A splice variant; VEGF-A_165_ uniquely binds to co-receptor neuropilin-1 (NRP-1) and significantly enhances its availability and binding to VEGFR-2 [12]. However, a very recent report indicated that NRP-1 mediates vascular permeability independently of VEGFR-2 activation [13]. Similarly, NRP-2 also binds VEGF-A_165_ with high affinity [14]. Preliminary investigations into NRP signaling suggested that due to the lack of kinase motif, NRPs were unable to mediate independent intracellular downstream signals upon ligand binding. Nonetheless, studies have indicated that NRP-1 regulates p130 (Cas) tyrosine phosphorylation through its intracellular domain during cell migration, which may explain its potential independent signaling mechanism [15,16]. Similar to VEGF members, PIGF can also bind both the neuropilins [17,18] and play important functions during angiogenesis. Interestingly, PIGF has been shown to specifically bind to VEGFR-1, with a possible indirect activation of VEGFR-2 through various mechanisms like transphosphorylation [19]. Readers are directed to a review by Hoeben and colleagues for a detailed description of all the VEGF isoforms and their corresponding receptors [4].

## 3. Role of Vascular Endothelial Growth Factors beyond Angiogenesis

Under physiological conditions, VEGFs regulate several developmental processes, including angiogenesis, lymphangiogenesis and neuronal development. However, they have extensive functions in several pathological processes affecting different organs in the human body. The current review will focus on some of the important pathologies associated with alterations in VEGF/VEGFR expression levels and not their mutations.

### 3.1. Rheumatoid Arthritis and Osteoarthritis

Rheumatoid arthritis (RA) is a persistent inflammatory disease of the joints whose detrimental effect often spreads beyond joints. RA is characterized by aberrant multiplication of synovial lining cells, causing enlargement of the synovial membrane. The synovia is often infiltrated with different immune cells like macrophages or lymphocytes [20]. Osteoarthritis (OA), commonly referred to as the “wear and tear” arthritis, is the most recurrent chronic non-inflammatory condition affecting the joints [21]. OA is characterized by degeneration of the hyaline cartilage that leads to severe stiffness and swelling, resulting in joint pain [22].

In an extensive review of the role of angiogenesis in arthritis, Walsh [23] described that the expression of VEGF-A_121_ isoform was notably increased in RA and OA conditions. The splice variants of VEGF-A; VEGF-A_121_ and VEGF-A_189_ were also detected in the osteoarthritic cartilage [24]. Importance of VEGF-A_165_, VEGFR-2, and NRP-1 has been well-studied in synovial angiogenesis occurring in RA [25,26]. Beyond its normal angiogenic function, VEGF-A has been shown to play crucial roles in chemotaxis for monocytes to enhance inflammatory response in the synovia [27]. Moreover, the induction of plasmin and matrix metalloproteinases through VEGF-A function is chiefly involved in degeneration of arthritic joints. Ballara et al. reported that the serum VEGF levels were significantly upregulated in patients with arthritis (RA, psoriatic, self-limiting arthritis and OA) when compared to healthy subjects [28]. Also, the serum VEGF levels were considerably greater in early RA patients in comparison to self-limiting arthritis patients. Another study by Lee and colleagues suggested that serum and synovial fluid VEGF levels were significantly greater in the RA patients than in the OA patients or healthy subjects [29]. Overall, these reports along with additional studies have described the importance and therapeutic benefit of VEGF/VEGFR-2 blockers and VEGF antagonists by ameliorating the symptoms of RA and OA [30,31,32,33,34]. Thalidomide (Apremilast, Celgene, Summit, NJ, United States) and paclitaxel (under the commercial name Paxceed™ Angiotech Pharmaceuticals Inc., Vancouver, BC, Canada) have already been tested in clinical trials for arthritic conditions. Interestingly, thalidomide has been shown to downregulate the expression of VEGF in human lung carcinoma cells [35]. However, the effect of paclitaxel on VEGF expression remains controversial, with one study indicating its ability to induce VEGF expression [36], while another demonstrating its VEGF-suppressive ability [37]. Although the Apremilast clinical study had to be terminated due to lack of efficacy [38], the long-term effectiveness of paclitaxel in RA patients is yet to be determined, in spite of its promising effect on cultured synovial cells from RA patients [39].

Similar to studies on VEGF-A, there have been numerous other studies investigating the roles of other VEGFs in RA and OA. Wauke et al. observed that VEGF-C protein was localized in several synovial lining cells, endothelial cells and stromal cells in RA synovial tissues [40]. Contrariwise, in synovial tissues obtained from OA patients, the VEGF-C protein was localized in the synovial lining cells and endothelial cells but with a lesser degree of expression [40]. Furthermore, the levels of the VEGF-C receptor; VEGFR-3 was significantly increased in RA than in OA. Immunostaining studies conducted on healthy and arthritic synovium indicated that VEGFR-3 ^+^ cells also co-expressed VEGFR-2 and other blood vessel markers [41]. Given the function of VEGF-C/VEGFR-3 in maintaining blood vessel fenestrations and synovial fluid formation, its ectopic expression in synovial microvessels could thus be partially explained. However, the exact functional implication and role in pathogenesis remain unclear. Also, VEGF-D was almost nonexistent in the synovial lining of RA patients. Finally, in a recent observational study, Kelly et al. showed a significant correlation between VEGF-C and VEGFR-3 expression with ultrasound assessment of synovitis in a cohort of early RA patients [42]. Taken together, all these studies imply the importance of VEGFs and their receptors in the progression of RA or OA; however, the mechanisms of action remain uncertain.

It is important to note that anti-VEGF/VEGFR therapies, particularly against cancer, have been associated with severe side-effects such as hypertension, gastro-intestinal toxicity, hypothyroidism, proteinuria, coagulation disorders, headache, dyspnea, fatigue, anorexia, stomatitis, diarrhea, neurotoxicity and thrombotic microangiopathy [43,44,45]. Additionally, repression of VEGF expression has been shown to produce significant defects in bleeding and clotting time in VEGF-repressed mice [46]. Due to the ability of VEGFR-2 to control blood pressure through regulation of nitric oxide synthase expression, anti-VEGFR-2 therapies could elevate the risk of hypertension significantly [47]. Therefore, careful measures need to be undertaken to ensure optimal and effective use of such therapies in such a way that the side-effects become manageable and pose less risk while treating pathological conditions.

### 3.2. Diabetes Mellitus and Associated Complications

Diabetes mellitus is a collection of metabolic disorders highlighted by hyperglycemia, resulting from impaired insulin secretion/action or both. This complex endocrine disease widely affects several organs in the body such as the eyes (retinas), kidneys, nervous system and vascular system. Primarily, VEGF-induced angiogenesis has been shown to play a major role in the pathogenesis of diabetic retinopathy through regulation of endothelial cell proliferation and vascular permeability [48]. However, beyond angiogenesis, both the VEGF-A_165_ and VEGF-A_165b_ isoforms have shown essential neuroprotective functions in the retina [49,50]. These studies suggest that anti-VEGF therapies could be damaging to the retinal and sensory neurons, opening new avenues for investigating novel therapies against diabetic retinopathy.

In a recent cross-sectional case-control study conducted in patients with type 1 diabetes mellitus or type 2 diabetes mellitus, significant associations were reported between single nucleotide polymorphisms (SNPs) in the *VEGF-C* gene and the development of diabetic retinopathy, indicating a possible functional role of VEGF-C in the pathogenesis of the disease [51]. However, the exact mechanistic roles of VEGF-C and other VEGF isoforms in diabetic retinopathy remain unknown. The role of VEGFs in the renal pathophysiology of diabetic nephropathy has been diverse and complex [8]. It is known that glucose can directly or indirectly increase the expression of VEGF-A and VEGF-A/eNOS-NO glomerular relationships are central to the pathogenesis of diabetic nephropathy [52,53]. Blockade of the renin-angiotensin, a critical player in elevating VEGF-A, demonstrated promising results to impede the development and progress of diabetic nephropathy. Furthermore, several studies investigated the relationship of VEGF-A with the insulin receptors and nephrin in the setting of diabetic nephropathy [54,55,56] and uncovered their significant pathogenic role. In Zucker diabetic fatty (ZDF) rats, renal or glomerular VEGF mRNA concentration rose early in the course of diabetes and remained elevated till 7 months [57]. Expression of VEGF and VEGFR-1/2 were increased 2-fold in retina and glomeruli from ZDF or insulin-resistant rats, indicating their potential as therapeutic targets [58]. In line with this study, Schrijvers et al. showed that a VEGF-neutralizing antibody prevented glomerular hypertrophy in the ZDF rats [59]. It is important to note that the course of VEGF expression during progression of diabetic nephropathy has been reported to be different in various animal models of type 2 diabetes [8]. Therefore, one needs to be careful when selecting the animal model for studying diabetes and interpreting the results.

Patients with diabetes have a two to five times greater risk of cardiovascular disease [60,61]. Two-fold reductions in VEGF and VEGFR-2 were reported in ventricles from diabetic patients compared to healthy controls [50]. A breakthrough study by Yoon et al. showed that the lack of VEGF expression might affect microvascular homeostasis in the myocardium and thus play a vital role in the pathogenesis of diabetic cardiomyopathy in streptozotocin-induced diabetic rats [62]. Subsequently, it was observed that redox imbalance and/or changes in VEGF expression were responsible for diabetic cardiomyopathy in a murine type 1 diabetes model [63]. More recently, Shida et al. investigated the effects of fluvastatin on diabetic cardiomyopathy and observed that the cardiac function was significantly improved through a reduction in myocardial oxidative stress and increase in VEGF levels [64]. A gene therapy study by Zeng et al. showed that Apelin gene therapy amended diabetic cardiomyopathy through a significant increase in sirtuin 3 and VEGF/VEGFR-2 expression via reducing oxidative stress and endothelial cell apoptosis [65]. Apelin is a bioactive peptide isolated from bovine gastric extract, characterized as an endogenous ligand of the human G-protein-coupled receptor APLNR (Apelin receptor) [66]. These studies document that VEGF-A/VEGFR-2 expression is differentially regulated in most of the diabetes-associated complications. Recent animal studies have suggested that VEGF-B signaling could play important roles in insulin resistance, lipid distribution, and metabolism in type 2 diabetes. In accordance, a study by Sun and colleagues demonstrated a clinical association/correlation of circulating VEGF-B with hyperlipidemia and organ damage in type 2 diabetic patients [67]. However, precise roles of other VEGF isoforms in diabetes-associated complications, if any, need to be investigated in the future.

It has been shown that the prevalence of vascular calcification (excess deposits of calcium mineral in the vessel) is increased in patients with diabetes [68]. Also, coronary artery calcification is now a recognized surrogate endpoint in the studies of diabetes with patients older than 30 years [69]. Interestingly, Yadav et al. reported a significant correlation of VEGF genetic polymorphisms to aortic calcification [70]. In the same study, age, hypertension, diabetes, dyslipidemia, and hyperhomocysteinemia were shown to be the contributing factors for aortic calcification in association with different VEGF genotypes. While demonstrating a direct role, Mikhaylova and colleagues showed that VEGF could induce mineralization in vascular smooth muscle cells [71]. Nonetheless, additional studies are required to further understand the cellular mechanisms in mediating vascular calcification.

### 3.3. Chronic Obstructive Pulmonary Disease

Chronic obstructive pulmonary disease (COPD) is a lung disease characterized by chronic bronchitis and emphysema. A report by Liebow and colleagues indicated that COPD-affected lungs demonstrate loss of interalveolar septa, making the septa appear extremely thin [72]. At the mechanistic level, Koyama et al. revealed that diminished VEGF levels were observed in the bronchoalveolar lavage fluid from smokers, which could possibly explain the thin and avascular nature of septa [73]. Moreover, VEGF expression was notably reduced in patients with idiopathic pulmonary fibrosis and sarcoidosis in comparison to nonsmokers, with an even further decrease in VEGF levels in smokers.

In another study, Kasahara et al. observed that the transcript and protein levels of VEGF and its receptor VEGFR-2 were diminished in lungs obtained from COPD patients. An important proposition emerged from the study that abnormal VEGF activity and endothelial dysfunction may play a crucial role in the pathogenesis of emphysema [74]. However, it still remains undetermined if reduced VEGF activity is the cause or effect of COPD. A breakthrough study by the same group showed that blockade of VEGFR-2 (using SU5416) in vivo leads to alveolar cell apoptosis and pathogenesis of emphysema in rats [75]. Lastly, a controlled cross-sectional analysis in COPD patients exhibited that oxidative stress-caused epithelial cell injury may lead to diminished VEGF expression in the lungs, which may subsequently result in the progression of COPD [76]. Overall, all these findings emphasize the prominence of VEGF signaling in the pathogenesis of COPD.

### 3.4. Endometriosis, Preeclampsia, and Ovarian Hyperstimulation Syndrome

Endometriosis is a medical condition characterized by pelvic pain due to an unusual manifestation of endometrial tissue outside the uterus [77]. It was believed that viable endometrial cells could give rise to endometriotic lesions when implanted into the peritoneal cavity through backward menstruation [78]. These ectopic lesions displayed a remarkable growth, leading to the progression of endometriosis. While examining the mechanisms, a direct correlation was observed between VEGF-A in the peritoneal fluid and endometriosis in women [79,80]. In the same year, McLaren and colleagues demonstrated that peritoneal fluid macrophages expressed steroid hormone receptors, VEGFR-1 and VEGFR-2 [80]. Moreover, these macrophages secreted VEGF-A under the influence of ovarian steroid hormones. VEGF receptors’ expression and the migratory reaction were notably greater in women with endometriosis, however, the potential of anti-angiogenic therapy against this disease warrants additional inquiry.

Preeclampsia is a pregnancy-related condition illustrated by high blood pressure accompanied by endothelial injury and dysfunction [81,82]. Several factors have been identified contributing towards endothelial damage such as deficiency of antithrombotic and vasodilator factors and increased concentrations of vasoconstrictor products, pro-thrombotic products, fibronectin concentrations and thrombomodulin. Additionally, neutrophil and platelet activation plays a pivotal role in vascular damage and endothelial dysfunction [83,84,85]. Although it is still unclear what prompts this chain reaction leading to endothelial dysfunction in preeclampsia, recent evidence has indicated that reduced fetoplacental perfusion and abnormal release of aforementioned factors in circulation may result in endothelial injury and consequent medical symptoms [86]. There have been controversies regarding the serum VEGF-A concentrations in women detected with preeclampsia [85,86] and future studies need to be undertaken to resolve the controversies surrounding VEGF-A and its diagnostic/therapeutic potential in preeclampsia. In addition to VEGF-A, VEGFRs have also been shown to have important roles in the pathogenesis of preeclampsia. It was demonstrated that VEGFR-1 expression was increased in patients with severe preeclampsia [87]. Interestingly, soluble VEGFR-1 (sVEGFR-1), an antagonist of VEGF and PIGF, was shown to upregulated in preeclampsia [88]. Moreover, delivery of sVEGFR-1 to pregnant rats induced hypertension, proteinuria, and glomerular endotheliosis, the classic lesion of preeclampsia [88], suggesting a plausible role of circulating sVEGFR-1 in its pathogenesis [89]. As a result, excessive circulating sVEGFR-1 splice variants have been proposed as predictive candidates causing the development of preeclampsia [90]. More recently, VEGFR-1 SNPs were associated with preeclampsia in a Philippine population [91]. Similarly, evidence indicated that differential activation of VEGFR-2 was associated with increased placental angiogenesis in early- as well as late-onset preeclampsia [92], however, mechanistic studies are warranted to pinpoint the exact function of VEGFR-2 in the pathogenesis of this clinical condition.

The ovarian hyperstimulation syndrome (OHSS) is another medical condition that occasionally affects the ovaries upon administration of fertility medicines to stimulate ovulation [93,94]. Enlargement of ovaries and enhanced vascular permeability are major contributing factors in the pathogenesis of OHSS. Several studies have reported elevated serum levels of VEGF during OHSS but it remains uncertain if this rise is the cause or effect of OHSS [95,96,97]. A case-control study reported that polymorphisms of VEGF and VEGF receptors were related to the incidence of OHSS [98]. Moreover, novel strategies inhibiting VEGF activity resulted in the reduction of alterations and severity associated with OHSS in rats [99,100]. Given the therapeutic benefit offered by VEGF antagonists, drugs targeting VEGF should certainly be contemplated for the prevention of OHSS [101].

### 3.5. Psoriasis

Psoriasis is a prevalent chronic skin disorder depicted by persistent red skin plaques frequently covered with loose scales, which may often be itchy and painful [102]. It is also characterized by infiltration of inflammatory cells and unusual growth of blood vessels that allows meeting the nutritional requirements of the hyperplastic epidermis. Detmar et al. determined that TGFα and EGF play essential roles in the synthesis of VEGF-A and its secretion by keratinocytes in psoriasis [103]. This finding was supported by the observed overexpression of TGFα and EGF receptors. Additionally, elevated levels of serum VEGF-A were observed in psoriasis patients [104]. However, additional research needs to be undertaken to investigate if this phenomenon is due to VEGF-A overproduction in the skin and subsequent leakage in the circulation or due to some genetic defect. Moreover, overexpression of VEGF in keratinocytes led to a significant increase in dermal angiogenesis and resulted in a psoriasis-like phenotype, emphasizing a central role of VEGF in the pathogenesis of skin disorders [105,106]. Neovastat (AE-941), an angiogenesis inhibitor has been shown to alleviate the clinical symptoms of patients suffering from psoriasis [107], indicating that anti-angiogenesis could be an effective strategy against psoriasis [108].

### 3.6. Neurodegenerative Disorders

The role of vascular growth factors in neurodegenerative disorders has intrigued researchers across the world. A seminal genetic study discovered that targeted aberrations in the promoter region of mice *Vegf* gene (VEGF∂/∂ mice) lead to adult-onset motoneuron degeneration, indicative of amyotrophic lateral sclerosis (ALS) [89]. In the same VEGF∂/∂ mice, although VEGF levels in the spinal cord were suppressed by about 25% and had no prominent flaws in angiogenesis, neural perfusion was notably reduced, resulting in chronic ischemia of motoneurons. A human study conducted in at least 3 European populations observed that low plasma levels of VEGF owing to faulty transcription and translation correlated with an elevated risk of ALS [109]. Additionally, the secondary effects of elevated VEGF accompanying the ischemic insult and inflammatory response associated with neurodegeneration could be detrimental as excess VEGF expression often results in hemangiomas, microvascular leakage, bleeding and edema, among others.

Several studies have investigated the neuroprotective effects of VEGF through its receptors VEGFR-2 and NRP-1 present in motoneurons [110]. It has been postulated in VEGF∂/∂ mice suffering from ALS that lack of VEGF survival signal may be responsible for the motoneuron degeneration [111]. In another study using VEGF∂/∂ mice, Lambrechts et al. demonstrated that these mice were abnormally vulnerable to continued paralysis post spinal cord ischemia and VEGF-A treatment protected mice against motoneuron death [109]. Furthermore, while clarifying the neuroprotective effect of VEGF signaling, VEGFR-2 overexpression delayed the motoneuron deterioration in another well-established ALS mouse model [112]. Confirming the neuroprotective role of VEGF, several in vitro studies have shown the pro-survival effects of VEGF against cell death when cultured motoneurons were exposed to hypoxia, oxidative stress, serum starvation [111], and superoxide dismutase-1 [113]. It is worthwhile to investigate these protective effects of VEGF in other neurodegenerative disorders, particularly due to the role of VEGF receptors in several neural cells [111,114,115] and the functional benefit offered by VEGF in different types of neurons [115,116], astrocytes [117], microglial cells [118] and Schwann cells [119].

### 3.7. Organ Fibrosis via Endothelial-to-Mesenchymal Transition

Matrix-yielding fibroblasts have multifactorial origins in fibrotic pathologies, with organ fibrosis via endothelial-to-mesenchymal transition (EndMT) being an important contributor towards fibrosis [120]. EndMT is an extreme form of cellular plasticity where endothelial cells lose typical cobblestone morphology and gain myofibroblast-like features and peculiar spindle-shaped morphology related to mesenchymal cells [121,122]. Although EndMT has been implicated in its physiological role during heart valve formation [123], recent evidence has indicated an additional role of this process in a variety of organ fibrosis disorders like renal [124], cardiac [125], pulmonary [126] and tumor fibrosis [127]. It has been shown that VEGF-A inhibits EndMT through VEGFR-2 signaling and has contrasting effects via VEGFR-1, where it promotes EndMT through decreasing the bioavailability of VEGF-A for VEGFR-2 [128]. Although the role of EndMT and VEGF has been investigated in kidney fibrosis [129], the exact roles and mechanisms of fibrosis in other organs remain undetermined. Furthermore, given the multifactorial role of VEGF in tumors, it remains important to investigate the VEGF-EndMT link in tumor fibrosis. Recently, our preliminary data indicated that cilia; the hair-like projection protruding from the cells, also regulated EndMT through modulation of VEGF-A expression in endothelial cells. Specifically, loss of endothelial cilia led to reduced expression of VEGF-A, leading to enhanced EndMT in vitro and increased organ fibrosis in vivo (unpublished data). Given the importance of EndMT and VEGF in different pathologies, future studies should focus on the causal role of this physiological process in great details.

VEGFs are also involved in the vascular pathology associated with most cases of Alzheimer’s disease, tumors, cerebral ischemia and coronary/peripheral artery disease; however, readers are directed to other reviews for detailed information regarding the same [130,131,132,133]. Additionally, VEGFs have emerged as important determinants and biomarkers for disease mortality and morbidity in several pathological conditions such as sepsis [134,135] or peripheral artery stenosis [136]. However, the potential role of VEGFs during initiation and progression of these conditions has not been studied till date.

## 4. Fibroblast Growth Factor

Interestingly, the first pro-angiogenic molecule to be ever recognized was the basic fibroblast growth factor (FGF2) [137]. Similar to the VEGF superfamily of growth factors, the FGF family is extensive and comprises of at least 20 factors that are categorized into 6 subfamilies depending on the sequence variation and phylogeny. Unlike most of the other growth factors, the widely studied FGFs; FGF1 and FGF2, do not contain the cytoplasmic signals for their extracellular expression. As a result, their role in angiogenesis had been uncertain for a long time until various studies indicated an alternative mechanism for their extracellular export [138]. Although FGFs display a great affinity to surface heparan sulfate proteoglycans (HSPGs), their biological function is mainly carried out via receptor tyrosine kinases, denoted FGFR1, 2, 3 and 4 [139,140] that arise through alternative splicing [141]. Extensive genetic studies have demonstrated the indispensable role of FGF signaling in angiogenesis and readers are directed to relevant research articles for further information [142,143,144,145]. It is important to note that FGF2 is a more potent angiogenic factor than VEGF-A [146] and hence could be a more viable anti-angiogenic target where FGF signaling outweighs the effect of VEGF signaling.

## 5. Role of Fibroblast Growth Factors beyond Angiogenesis

The family of FGFs regulates a plethora of developmental processes, including brain patterning, branching morphogenesis, and limb development. Different FGFs are involved in diverse disease conditions. It is beyond the scope of current review to mention disease association of each FGF isoform, however, the most relevant and well-studied FGF-disease links have been discussed. The detrimental genetic disorders that arise from FGF and FGFR mutations have been reviewed elsewhere in detail [147,148].

### 5.1. Non-Alcoholic Fatty Liver Disease

Non-alcoholic fatty liver disease (NAFLD) is a broad term encompassing several metabolic liver diseases like fatty liver (steatosis), non-alcoholic steatohepatitis (NASH) and liver cirrhosis [149,150]. The incidence of NAFLD is quite common with most patients demonstrating none to very few symptoms. However, in some cases of an extreme form of the disease (e.g., NASH), the excessive fat accumulation could cause hepatic inflammation and damage the liver. In its more serious manifestation, NAFLD can cause complete liver failure. Several risk factors such as obesity, dyslipidemia (abnormal amount of lipids in the blood) and insulin resistance are associated with NAFLD [151,152,153].

Hanaka and colleagues [154] observed a noteworthy function of FGF5 and role of diet in the progression of NASH (specifically hepatic fibrosis) using *Fgf5*-null mice. However, future investigations are necessary to elucidate the mechanisms linking FGF5, diet, and NASH. Remarkably, in patients with NASH and hepatic steatosis, serum FGF21 concentrations were notably elevated and correlated with the degree of steatosis, indicating its great utility as a serum biomarker for diagnosis [155,156]. Interestingly, in patients with severe NAFLD, FGF21 was significantly decreased owing to the damaged liver [157], suggesting a possible correlation between FGF21 with the degree of disease. In mouse models for NASH, the serum FGF21 levels and its expression in the liver were reported to be drastically high [158,159]. In diet-induced obese mice suffering from NAFLD, Xu et al. [158] described that administering FGF21 reduced body weight and whole-body fat mass, blood glucose, insulin and lipid levels, and reversed hepatic steatosis. Very recent studies have suggested that modulation of miR-49 [160] and miR-212 [161] offered a protective function against NAFLD by targeting FGF21. Taken together these results indicate that FGF21 plays a vital role in multiple metabolic syndromes and has the potential to become an influential therapeutic option to alleviate the symptoms of NAFLD.

Contrastingly, in pediatric NAFLD, serum FGF21 and FGF19 are inversely associated with hepatic damage and these findings may have crucial associations for pinpointing the exact mechanisms of NAFLD progression in adults versus children [162].

### 5.2. Vascular Calcification

Vascular calcification is a condition where large deposits of calcium mineral are progressively found in main arteries, reducing their elasticity and disturbing the cardiovascular hemodynamics [163,164]. This results in substantial morbidity and mortality mostly in individuals aged >60 years [165]. In subjects from the Prospective Investigation of the Vasculature in Uppsala Seniors (PIVUS) study, higher serum FGF23 quantities were independently related with weakened vasoreactivity and augmented arterial stiffness [166]. Furthermore, Ozkok et al. reported a correlation between the coronary artery calcification score (CACS) and FGF23, indicating that FGF23 might have a substantial role in the development of vascular calcification, particularly during the initial steps in hemodialysis patients [167]. A significant association between FGF23 and vascular calcification was also reported in peritoneal dialysis patients [168]. Furthermore, in a cross-sectional study in patients with suspected coronary artery disease, serum FGF23 levels were associated with coronary calcification. Interestingly, in patients who underwent CACS, serum FGF23 levels were associated with vascular calcification, hinting a possible involvement of FGF23 in the formation of atherosclerotic process [169]. Finally, in a recent study with hemodialysis patients, high-flux hemodialysis benefitted the patients through a reduction in the serum FGF23 levels and subsequent reduction in vascular calcification [170].

Overall, these studies indicate a vital role of FGF23 in vascular calcification, a process commonly associated with chronic kidney disease (CKD) and coronary artery disease. Mechanistically, the FGF23/klotho axis has been comprehensively investigated in the vascular calcification process and metabolic disorders involving the same [171]. Additionally, Zhu et al. revealed the essentiality of the ERK1/2 signaling pathway for FGF23 in promoting vascular smooth muscle cell calcification [172].

### 5.3. Cardiac Hypertrophy

Cardiac hypertrophy is an abnormal thickening of the myocardium, resulting in smaller-sized left and right ventricles of the heart and subsequent impaired heart function. Studies have indicated a connection between circulating FGF23 levels and cardiovascular pathologies such as left ventricular hypertrophy. Left ventricular hypertrophy is highly predominant in CKD settings, therefore, such associations have been primarily investigated in patients with CKD [173]. Furthermore, recent studies have identified activation of FGFR4 as the primary event facilitating the effects of FGF23 on left ventricular hypertrophy, thus postulating novel mechanistic insights and therapeutic targets [174,175,176].

Reports have indicated that circulating concentrations of calcium, phosphorus, and 1,25-dihydroxy vitamin D3 (1,25(OH)2D3) are not only linked with vascular calcification but also with ventricular hypertrophy [177]. Van Ballegooijen et al. showed that increased parathyroid hormone (PTH) levels were associated with left ventricular mass and cardiac troponin T in patients with chronic kidney disease [178] and that FGF23 inhibited its expression and production. Interestingly, 1,25(OH)2D3 stimulated the expression of FGF23 mRNA in osteoblast cell cultures, suggesting a physiologic role of FGF23 to act as a counter regulatory phosphaturic hormone for maintaining phosphate homeostasis in the presence of vitamin D [179]. FGF23 has a dual role in regulating calcitriol (active metabolite of vitamin D) synthesis. Firstly, it downregulates expression of *Cyp27b1* gene that translates into 1-alpha-hydroxylase, the enzyme responsible for switching 25(OH)D3 to 1,25-(OH)2D3. Secondly, it also activates the degradation pathway through upregulation of 24-hydroxylase, the enzyme disabling 1,25(OH)2D3 [179,180]. Thus, due to the complex roles of calcium, phosphorus, vitamin D and PTH in cardiovascular pathologies, and the ability of FGF23 to regulate each, makes FGF23 a central player and a therapeutic target in cardiac dysfunction.

Some of the other FGFs have also been shown to play pivotal roles in cardiac repair. For example, FGF2 acts as an essential watchdog of cell proliferation, angiogenesis, collagen production, cardiomyocyte hypertrophy, scar contraction and left ventricular contractile function during infarct repair [181]. In a breakthrough discovery, Faul and colleagues showed a causal function of FGF23 in the pathogenesis of left ventricular hypertrophy [182]. They observed that recurrently elevated FGF23 levels via intramyocardial and intravenous injections of FGF23 in mice, led to high rates of ventricular hypertrophy and mortality. An extensive review by Itoh and Ohta described the pathophysiological roles of FGF signaling in the heart [183]. Mechanistically, FGF2 promotes cardiac hypertrophy and fibrosis through MAPK signaling via the activation of FGFR1c [183,184]. Contrastingly, another FGF isoform; FGF16 showed preventive roles since it competed with FGF2 for binding to the FGFR1c [185]. Also, FGF21 blocked cardiac hypertrophy by activating MAPK signaling through the activation of FGFR1c along with co-receptor βKlotho [186]. Moreover, FGF23 provoked cardiac hypertrophy through calcineurin/NFAT signaling without αKlotho co-receptor [182,187]. While high molecular weight isoform of FGF2 induced hypertrophy through ERK activation, FGF23 depended profoundly on PLC-γ-calcineurin-NFAT activation [188,189]. Together these results indicate that FGFs are involved in cardiac remodeling via distinctive molecular mechanisms.

### 5.4. Atherosclerosis

Atherosclerosis is characterized by narrowing of the arteries due to building up of plaque inside them [190]. It is a common cause of heart attacks, strokes, and peripheral vascular disease. Chow et al. demonstrated for the first time that increased serum FGF21 concentrations positively correlate with carotid atherosclerosis in a group of Southern Chinese subjects [191]. A cross-sectional study by Semba et al. reported an association between FGF21 levels and hypertension in 744 community-dwelling adults that had participated in the Baltimore Longitudinal Study of Aging [192]. In an interesting study involving aerobic exercises, Yang and colleagues reported that a 3-month combined exercise program reduced the FGF21 quantities as well as arterial rigidity in overweight Korean women [193]. A recent study examining the molecular mechanisms for progression of atherosclerosis showed that the extent of coronary atherosclerosis strongly correlated with the loss of endothelial FGFR1 expression, activation of endothelial TGF-β signaling and the degree of EndMT [194].

Furthermore, FGF/TGF-β signaling cross-talk was discovered as an important regulator of smooth muscle cell phenotypic switch and a major contributor to atherosclerotic plaque growth in mice [195]. Subsequent examination of human coronary arteries with varying grades of atherosclerosis exhibited a robust correlation between the activation of FGF signaling, loss of TGFβ signaling and augmented disease severity. In a rat model of atherosclerosis, FGF21 markedly amended the atherosclerotic symptoms through a reduction in serum levels of total triglyceride, low-density lipoprotein cholesterol, and total cholesterol, accompanied by an increase in the serum levels of high-density lipoprotein cholesterol HDL-C [196]. In vitro, FGF21 prevented cell apoptosis by inhibiting the MAPK pathway. Lastly, a plethora of recent studies has indicated the crucial role of FGF23 in the pathogenesis of atherosclerosis, implicating its utility as a biomarker as well as a therapeutic target [177,197,198,199,200,201,202,203].

### 5.5. Chronic Kidney Disease (CKD)

CKD is referred to as the continuing loss of kidney function. Under such conditions, the normal function of the kidneys to filter-out wastes and surplus fluids from blood and excrete in the urine is severely affected. During the end stage of this disease, threatening levels of fluid, creatinine, electrolytes and wastes could accumulate in the body [204]. Although FGFs gained much attention due to their clinical relevance in patients with CKD, a majority of the studies were focused on FGF23 and its role in CKD. Studies investigating the function of FGFs (primarily FGF23) as diagnostic markers, causal factors, and therapeutic targets are briefly mentioned in Table 1.

### 5.6. Lung Disease

In a comprehensive review, Hines and Sun highlighted the importance of FGF, Notch, and Wnt signaling pathways in the development of lung and lung repair pathways [216]. Indeed, after naphthalene injury in mice, parabronchial smooth muscle cells secreted FGF10 to activate Notch signaling and induced Snail expression to induct repair process in the injured lung [217]. Furthermore, FGF10 expression was amplified in smooth muscle cells in the presence of Wnt signaling. FGF10 signaling consequently augmented the Notch pathway leading to a transient epithelial-mesenchymal transition (EMT) in the airway.

Investigating the contributions of other FGF isoforms, Coffey et al. demonstrated that FGF9 protein was aberrantly overexpressed in areas of active cellular hyperplasia, metaplasia, and fibrotic expansion of idiopathic pulmonary fibrosis (IPF) lungs [218]. Similarly, elevated expression of FGF1/FGFRs in the pathogenic regions of IPF suggested that aberrant FGF1-FGFR signaling may contribute to the pathogenesis of lung fibrosis [219]. Additionally, FGF1 was able to revert TGFβ1-induced EMT through MAPK/ERK kinase pathway in alveolar epithelial-like cell lines [220]. FGF strong inhibitor (capable of binding FGFs and blocking FGFR signaling), showed a robust potential to alleviate bleomycin-induced lung fibrosis through inhibiting the expression of α-smooth muscle actin and collagen deposit. Contrastingly, transplantation of FGF7-lentivirus-transduced hematopoietic stem cells diminished bleomycin-induced lung injury, showing the potential of cell-based gene therapy in the lungs [221]. FGF2 has been shown to be required for epithelial recovery, but not for pulmonary fibrosis, in response to bleomycin, which calls for more studies to explore the role of FGF2 as a profibrotic growth factor in vivo [222]. A recent study by Joannes and colleagues demonstrated that FGF9 and FGF18 foster survival and migration in IPF, and prevent in vitro myofibroblast differentiation of human lung fibroblasts [223]. Finally, several small molecule inhibitors/drugs/products have been tested in pre-clinical and clinical studies for their efficacy against lung diseases through modulation of FGF signaling [224,225,226,227,228,229].

### 5.7. Cutaneous Inflammation

Inflammation of the skin may arise due to a variety of reasons such as radiation, thermal burns or skin injuries/diseases, and in such instances effective wound healing is fundamental for the maintenance of skin integrity. Several studies have indicated the contribution of FGF signaling pathway in skin repair processes. One such study revealed that loss of the IIIb splice variants of FGFR1 and FGFR2 in keratinocytes led to a gradual loss of skin appendages, cutaneous inflammation, keratinocyte over proliferation, and epidermal hyperplasia (thickening of the skin; acanthosis) [230]. Furthermore, FGFs 7, 10, and 22 that stimulate both FGFR1b and 2b, are abundantly expressed in both normal and wounded skin and are critical for maintaining the epidermal wall. Depending upon the FGF isoform, receptor activation can either be autocrine (FGF22) or paracrine (FGF7 and FGF10) [230,231].

In an attempt to understand the mechanisms of hair follicle regeneration, Gay et al. discovered that γδT-cells (a type of T lymphocytes) were important sources of FGF9, which is essential for hair follicle regeneration after wounding [232]. In a mouse model, they found that FGF9 from γδ T cells activated Wnt expression and consequent Wnt activation in fibroblasts. Furthermore, these activated fibroblasts then express FGF9, thus amplifying Wnt activity through a feedback loop, leading to regeneration of hair follicles. Werner et al. showed that FGF2, FGF5, and FGFR1/2 were significantly upregulated upon skin injury, thus indicating the prominence of FGF signaling in wound healing [233]. Intriguingly, a peptide mimetic of FGF2 (FGF-P) protected against acute radiation-induced injury and showed great potential as a future therapeutic option against thermal burns, ischemic wound healing, and tissue regeneration [234]. Finally, an antibacterial protein from the venom of *Crotalus adamanteus* (diamondback rattlesnake) toxin-II (CaTx-II, of rattlesnake) when utilized for treating wounded mice exhibited noteworthy wound closure and complete healing, accompanied by upregulation in FGF2 levels and other wound healing associated cytokines [235]. Taken together these studies emphasize the central function of effective FGF signaling in the inhibition of cutaneous inflammation.

### 5.8. Alzheimer’s Disease

Alzheimer’s disease is a progressive mental condition due to widespread deterioration of the brain that is often associated with impaired memory. The nerve growth factor (NGF) and brain-derived neurotrophic factor (BDNF) have been widely studied with respect to their role in the pathogenesis of Alzheimer’s disease [236]. Remarkably, FGF2 demonstrates notable functional similarities with neutrophins. For example, FGF2 prevented the neuronal damage and neurofibrillary tangles-like antigenic changes post-glucose deprivation in the brain [237]. Interestingly, FGF2 interacted with BDNF to activate Akt and ERK to offer neuroprotection [238,239]. Several studies reported an induction and binding of FGF2 to the plaques and neurofibrillary tangles in brains affected by Alzheimer’s disease [240,241,242], and in cerebrospinal fluid (CSF) obtained from Alzheimer’s disease patients [243]. Cummings and colleagues showed that FGF2 attracts neurites into plaques and subsequently a damaged neurite may stimulate FGF2 production resulting in further neuritic attraction [244]. While elucidating the distribution of FGFR1 in the cortex and hippocampus of patients with Alzheimer’s disease, immunoreactivity of the FGFR1 (that binds FGF1/2) was augmented in responsive astrocytes, neighboring senile plaques in patients with Alzheimer’s disease when compared to age-matched controls [245].

At the molecular level, it has been determined that the microtubule-related Tau protein is more phosphorylated at certain residues in Alzheimer’s disease paired helical filaments than in normal brain [246,247]. FGF2 stimulation resulted in augmented Tau phosphorylation in neuronal cultures [248] via elevated expression of Tau kinase GSK-3β and Tau protein [249,250,251]. A recent study by Hong et al. showed that the treatment of rats with Puerarin (an isoflavone used for treating Alzheimer’s disease) could considerably improve behavioral performance and the heightened neurogenesis and Tau protein phosphorylation possibly via the FGF2/GSK-3 signaling pathway [252]. A novel synthetic compound that mimics the neuroprotective properties of FGF2 (SUN11602) perfected memory and learning deficits in the hippocampal-lesioned rats through prevention of neuronal death and/or promotion of neurite outgrowth, confirming the utility of such agents for treating neurodegenerative diseases such as Alzheimer’s disease [253]. The presence of senile plaques is one of the key pathological hallmarks of Alzheimer’s disease. These plaques are mainly comprised of potentially toxic amyloid beta-peptide (Abeta) that is produced from a family of Abeta-containing precursor proteins (APP). Interestingly, analogous to NGF, FGF2 acts on the APP promoter, enhances APP transcription and the secretion of sAPP [254,255]. Moreover, when cultures of microglia and astrocytes were exposed to a synthetic homolog of Abeta, they synthesized more FGF2 and promoted the proliferation and morphological alteration of microglia that may contribute to the process of plaque growth [256]. Also, double transgenic mice overexpressing APP and FGF2 exhibited a greater mortality rate than mice expressing APP only, indicating that FGF2 transgene overexpression could augment the deleterious effects of APP [257].

Lastly, the FGF1 levels in serum and CSF were significantly higher in Alzheimer’s disease patients [258]. Although FGF1 may be involved in the pathophysiology of Alzheimer’s disease similar to FGF2, more studies need to be commenced to deduce the exact molecular signaling pathways.

### 5.9. Corneal Fibrosis

The cornea is the transparent layer in front of the eye, which is composed of an extremely organized group of cells and proteins. The corneal endothelium plays a vital function in maintaining corneal hydration and corneal clearness. Unlike most of the other cells in the human body, adult corneal endothelial cells (CECs) are mitotically inactive, being stopped at the G1 phase of the cell cycle [259]. Although mitogenic FGF2 is present in the Descemet’s membrane (membrane between the corneal proper substance and endothelium) [260], corneal endothelium maintains its anti-proliferative nature throughout its lifetime. However, upon corneal injury, corneal endothelium employs two diverse wound repair pathways. Firstly, via the regenerative pathway, CECs are substituted by migration and spreading of surviving endothelial cells. Secondly, the non-regenerative pathway (or fibrosis), by which altered endothelial cells recommence multiplication, change their cellular morphology and collagen phenotypes, resulting in the generation of an atypical fibrillar extracellular matrix (ECM). A clinical manifestation of such an event is the development of a collagenous retrocorneal fibrous membrane (RCFM) between Descemet’s membrane and the corneal endothelium that causes impaired vision [261].

While unraveling the molecular mechanisms of RCFM formation, studies revealed that FGF2 exerts a dominant role in EndMT that may contribute to corneal fibrosis significantly. Specifically, FGF2 demonstrated a regulatory role in cell cycle progression through degradation of p27Kip1 (p27), a negative controller of the G1 phase of the cell cycle [262,263]. Furthermore, FGF2 increased the steady-state concentrations of α1(I) collagen mRNA by stabilizing the message and subsequently secreting type I collagen into the extracellular space [264]. The same study concluded that FGF2 mediated corneal EndMT through the action of phosphatidylinositol (PI) PI3-kinase. In accordance with EndMT characteristics, FGF2 also induced phenotypic switching where cells changed from atypical cobblestone/polygonal shape to a spindle-shaped/fibroblastic morphology [263]. In vitro, interleukin-1β (IL-1β) induced FGF2 through the PI3-kinase and p38 pathways in CECs and acted as a regulatory switch for FGF2-induced EndMT [265]. Similar findings were reported in vivo when it was discovered that polymorphonuclear leukocytes that intrude the anterior chamber are the main source of IL-1β, which successively facilitates FGF2 production in the corneal endothelium [266]. Together these results provide a primary mechanistic link between injury-associated inflammation and FGF2-induced EndMT.

In a rabbit model of carbon dioxide laser injury, FGF10 significantly abridged inflammation, stromal edema, fibrosis and corneal neovascularization, indicating a major role of FGF10 in the regulation of corneal epithelial wound healing [267].

## 6. Angiopoietins

Angiopoietins are a family of vascular growth factors, comprising of three structurally linked proteins, termed Angiopoietin-1 (Ang-1), Angiopoietin-2 (Ang-2), and Angiopoietin-3/4 (Ang-3/4) [268]. Ang-1 and Ang-2 remain the most studied with respect to their function during physiological development. These ligands are considered crucial for vascular differentiation through angiogenesis and also participate in the maintenance of blood vessels as well as lymphatic vessels in late gestation and in adult animals [268,269,270]. Ang-1 and Ang-2 bind to the Tie2 (tyrosine kinase with Ig and EGF homology domain 2) receptor tyrosine kinase, whose expression is specific to the blood endothelial cells [271,272]. Interestingly, Ang-1 activates Tie2 as observed via augmented tyrosine phosphorylation of Tie2, while Ang-2 inhibits receptor activation and Ang-1-induced phosphorylation by competitively binding to the receptor. The homologous receptor Tie1 is relatively less well-studied. Due to its expression on developing endothelia, it is postulated that Tie1 inhibits Ang-1-induced Tie2 phosphorylation, thereby delivering a regulatory mechanism for signaling during early development [273].

## 7. Role of Angiopoietins beyond Angiogenesis

Endothelial dysfunction is associated with many pathological states. Studies focusing on the angiopoietins in dysfunctional blood vessels have provided further data supporting their multiple biological roles in angiogenesis. Some of the major findings involving the role of angiopoietins beyond angiogenesis have been summarized in Table 2 and Table 3. We have included the studies showing the role of angiopoietins in vascular calcification, diabetic retinopathy, diabetic nephropathy, chronic kidney disease, inflammatory bowel disease, pulmonary hypertension, and psoriasis. Additionally, angiopoietins have important causal or diagnostic roles in arthritis [274,275,276,277,278,279,280,281], psoriasis [282], and infertility [283,284,285]. For a detailed role of angiopoietins in ischemia, readers are directed to several excellent reviews [286,287,288].

Furthermore, angiopoietin-like proteins (ANGPTLs) are a family of proteins structurally similar to the angiopoietins that are involved in a plethora of physiological and pathophysiological processes. Eight ANGPTLs have been discovered (ANGPTL1 to ANGPTL8) to date. Several elegant reviews exist describing their function in various diseases [289,290,291,292,293,294].

## 8. Conclusions

**Figure 2:**
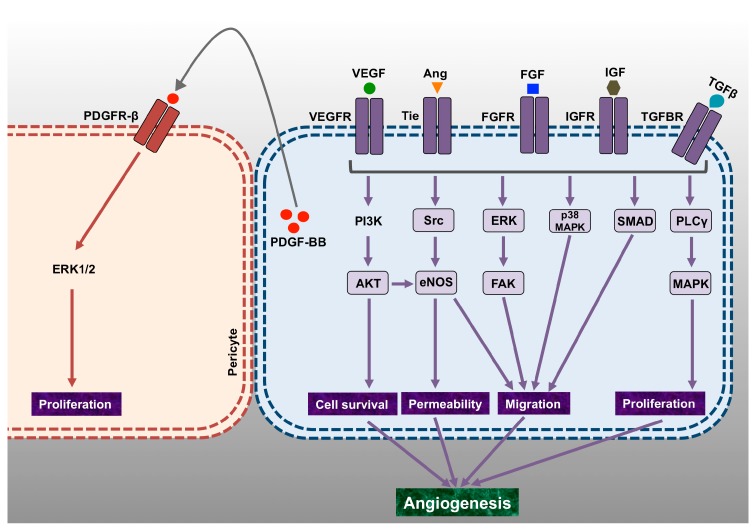
Overview of the major signaling pathways that mediate proliferation, vascular permeability, cell migration and cell survival, leading to angiogenesis. The schematic diagram represents some of the most important angiogenic growth factors and their signaling via respective cell-surface receptors that leads to proliferative, invasive, vasodilatory and permeability alterations fundamental for cell invasion and angiogenesis. Some of these pathways that are functional during normal physiological conditions can also participate in several pathophysiological processes. PDGFR: Platelet-Derived Growth Factor Receptor; IGF: Insulin-Like Growth Factor; ERK: Extracellular Signal–Regulated Kinase; MAPK: Mitogen-Activated Protein Kinase; PI3K: Phosphatidylinositide 3-Kinase; PLC: Phospholipase C; eNOS: Endothelial Nitric Oxide Synthase; FAK: Focal Adhesion Kinase.

Numerous studies have documented the indispensable role of VEGFs, FGFs, and angiopoietins in angiogenesis and related disorders. Functions in vascular permeability, mitogenesis, endothelial cell proliferation, vessel maturation and lymphangiogenesis have been extensively described (Figure 2). Additionally, these growth factors are also actively involved in other angiogenesis-related cellular processes like cell adhesion, cellular differentiation, and recruitment of inflammatory cells.

Substantial research has established the myriad pathological processes that involve angiogenic signaling. Therefore, contemplation of broader biological functions of these factors may elucidate the unusual and distinctive roles played by these angiogenic growth factors during disease initiation and progression. These mechanistic insights would lead to the discovery of novel therapies involving recombinant proteins, small molecules, antibodies and gene therapy in the future. Pathological conditions like arthritis, diabetes, kidney disorders, psoriasis, pulmonary and cardiovascular complications and Parkinson’s disease have been treated by angiogenic growth factor-based therapies in several pre-clinical studies. Although the therapeutic potential has been demonstrated in adult humans, the considerable task remains on hand to prevent and treat these diseases at birth. Further mechanistic studies unraveling the intricate roles played by these growth factors will allow to target disorders, both in utero and post-natally. Owing to their multifactorial function in various angiogenesis-independent processes, we believe that it will be more precise to consider these ‘angiogenic factors’, as wide-ranging ‘endothelial factors’. With this comprehensive description of biological activities beyond angiogenesis, these growth factors may emerge as important biomarkers and therapeutic targets in more, as yet unknown pathological conditions.

## Figures and Tables

**Table 1:** Diverse roles of fibroblast growth factor (FGF) isoforms in diseases of the kidney.

Author and Year	Target Studied	Study Details	Main Findings and Conclusions
Urakawa, et al. (2006) [205]	FGF23	In vitro: HEK293 cells, peak rapid cells (derived from HEK293 cells), CHO cells, and L6 myogenic cells	- Klotho was crucial for endogenous FGF23 function and klotho by itself was unable to mediate intracellular signaling - Membrane Klotho in concert with the FGF23 receptor increased the FGF23 receptor specificity and mediated the activity of Klotho-dependent FGF23 - The combined action of Klotho and FGFR1(IIIc) constituted the FGF23 receptor
		In vivo: Klotho-deficient (kl/kl) mice, Klotho-insufficient (kl^/+^) mice, Fgf23(^+/−^) and Fgf23(^−/−^) and *Fgf23*(^+/−^)(kl^+/−^) mice	
Fliser, et al. (2007) [206]	FGF23	Human studies: 227 white patients who were between 18 and 65 years of age and had non-diabetic CKD and various degrees of renal impairment were recruited. The primary cause of kidney disease was glomerulonephritis in 97 (biopsy-confirmed in 90) patients, adult polycystic kidney disease in 37 patients, interstitial nephritis in 24 patients, other types of kidney disease in 43 patients, and unknown in 26 patients	- In the baseline cohort, a substantial inverse correlation was noted between glomerular filtration rate and levels of both c-terminal and intact FGF23 - About 65 patients recorded a doubling of serum creatinine and/or fatal kidney failure and had a considerably lesser glomerular filtration rate at baseline. Contrastingly, they had higher levels of intact parathormone, serum phosphate and c-terminal and intact FGF23 - Both c-terminal and intact FGF23 independently projected the advancement of CKD
Isakova et al. (2011) [207]	FGF23	Human studies: 3879 participants with CKD stages 2 through 4 who were enrolled in the Chronic Renal Insufficiency Cohort between June 2003 and September 2008	- Greater concentrations of FGF23 were independently linked to a higher risk of death - Increased FGF23 was an independent risk factor for end-stage renal disease in patients with somewhat well-preserved kidney function and for mortality within the range of CKD
Gutiérrez, et al. (2008) [208]	FGF23	Human studies: FGF23 levels were assessed in 10,044 patients who were starting hemodialysis treatment Mortality was analyzed in a nested case-control sample of 200 subjects who died and 200 who survived during the first year of hemodialysis treatment	- Elevated FGF23 levels were related to an increased risk of mortality when studied either on a continuous scale or in quartiles - Elevated FGF23 levels independently correlated with mortality among patients who were commencing hemodialysis treatment
Isakova et al. (2011) [207]	FGF23	Human studies: 67 adults undergoing peritoneal dialysis for treatment of ESRD were studied	- FGF23 levels were associated with serum phosphate, residual kidney function, phosphate clearance and dialysis vintage - Higher FGF23 was linked with loss of residual renal function and larger dialysis vintage independent of population distribution, laboratory values, peritoneal dialysis method and suitability, and treatment with vitamin D mimics and phosphate binding agents - FGF23 could be a steadier indicator of phosphate uptake in ESRD than PTH or serum phosphate
Isakova et al. (2011) [207]	FGF23	Human studies: FGF23 was measured in baseline samples from 3879 patients in the Chronic Renal Insufficiency Cohort study, which is a distinct group of patients with CKD stage 2–4	- Mean serum phosphate and median PTH quantities were in the usual range, but median FGF23 was distinctly amplified than in healthy subjects and boosted considerably with diminishing eGFR - Increased FGF23 is a common manifestation of CKD that shows up earlier than elevated phosphate or PTH
Pavik et al. (2013) [209]	FGF23	Human studies: 87 patients at various stages of CKD unaffected by polycystic kidney disease nor having undergone kidney transplantation, were registered	- Although soluble klotho and 1,25-dihydroxy vitamin D(3) levels decreased and FGF23 levels increased at early CKD stages, PTH levels were elevated only at more progressive stages of CKD
Gutiérrez, et al. (2005) [208]	FGF23	Human studies: 80 patients from across the spectrum of CKD were enrolled in the study	- There was a negative correlation between FGF23 and PTH with eGFR, while calcitriol levels were linearly correlated with eGFR - Elevated Fe(PO_4_) levels correlated with reduced eGFR, and both increased FGF23 and PTH were independently linked to increased Fe(PO_4_) - Although higher FGF23 and lower 25(OH)D3 levels were independent predictors of reduced calcitriol, the effects of calcitriol levels on kidney function and hyperphosphatemia were totally abolished when corrected for FGF23 - FGF23 levels rose early in CKD prior to the development of serum mineral anomalies and were independently associated with serum phosphate, Fe(PO_4_), and calcitriol insufficiency
Floege et al. (1995) [210]	FGF2	In vivo: PHN was induced in male Sprague Dawley rats	- After treatment with FGF2, podocytes of PHN rats exhibited substantial escalations in mitoses, pseudocyst development, foot process retraction, focal detachment from the glomerular basement membrane, and desmin appearance - FGF2-injected PHN rats had increased glomerulosclerosis in comparison to control animals. Also, FGF2 provoked proteinuria and podocyte injury in rats infused with 10% of the routine PHN-serum dose - FGF2 augmented podocyte damage, resulting in amplified glomerular protein permeability and faster glomerulosclerosis
Guan et al. (2014) [211]	Klotho and FGF2	HK-2 cells, a proximal tubular cell line, and the normal rat kidney blast cell line NRK-49 F HK-2 cells, a proximal tubular cell line, and the normal rat kidney F	- In vitro, FGF2 produced tubulo-epithelial plasticity and reduced klotho expression. Recombinant klotho protein could constrain FGF2 activity - The FGF2 effects were mediated via ERK1/2 that were repressed by klotho. Klotho also inhibited FGF2-mediated fibroblast proliferation - Increased FGF2 and reduced klotho was associated with UUO-induced renal fibrosis in WT mice. FGF2^−/−^ mice mostly preserved klotho expression and exhibited only slight renal fibrosis after UUO injury - Intake of klotho protein in UUO mice notably abridged renal fibrosis, accompanied with a striking reduction in FGF2 production and activity
		In vitro: HK-2 cells, a proximal tubular cell line, and the normal rat kidney fibroblast cell line NRK-49 F	
		In vivo: Male C57BL/6 mice, WT mice, and FGF2-knockout (FGF2^−/−^) mice. UUO was performed to induce renal fibrosis	
Rossaint et al. (2016) [212]	FGF23	In vitro: Hematopoietic stem cells were isolated from WT mice	- Although leukocyte recruitment into inflamed areas and host defense is deterred by CKD, FGF23 nullification during CKD in mice reinstated leukocyte recruitment and host defense - FGF23 inhibited chemokine-activated leukocyte capture on the endothelium, and reduction in FGFR2 on PMNs salvaged host defense in these mice - In vitro, FGF23 inhibited PMN adhesion, arrest under flow, and transendothelial movement. Additionally, FGF23 binding to FGFR2 counteracted selectin- and chemokine-stimulated β2 integrin activation on PMNs via activation of PKA and impeding the activation of the small GTPase Rap1
		In vivo: Chronic kidney failure in mice was induced by 5/6-nephrectomy in male C57BL/6 mice *E. coli*-induced pneumonia was established	
Rossini et al. (2005) [213]	FGF1	Human studies: Formalin-fixed, paraffin-embedded kidney tissues from normal control kidneys, PLN, NPLN, AIN, and from transplant nephrectomies with acute rejection and CAN were included in this study	- FGF1 was detected in mesangial cells, glomerular endothelial, visceral, and parietal epithelial cells in normal kidney tissues. FGFR1 staining displayed a comparable pattern but was also detected in tubular epithelium, arterial endothelium, and smooth muscle - FGF1 expression was augmented over normal in glomerular parenchymal cells in podocytes and parietal epithelial cells only in CAN. Intruding glomerular and interstitial inflammatory cells in affected glomeruli also showed FGF1 and FGFR1 expression - Increased FGFR1 but not FGF1 was observed in tubular cells in diseased kidneys vs. normal kidneys
Silswal et al. (2014) [214]	FGF23	In vitro: Primary mouse endothelial cells were isolated from aorta by enzymatic digestion	- All four subtypes of FGF receptors were found in male mouse aortae - Exogenous FGF23 neither stimulated contraction of aortic rings nor relaxed the rings precontracted with prostaglandin F2α - Pretreatment with FGF23 led to a ~36% prevention of endothelium-relied relaxation stimulated by acetylcholine (ACh) in precontracted aortic rings. This was inhibited by the FGFR antagonist - Col4a3−/− CKD mice exhibited exceedingly raised serum FGF23 levels and had compromised endothelium-dependent relaxation. They also showed abridged nitrate production as compared to WT - Exogenous FGF23 caused increased superoxide levels in endothelial cells and aortic rings
		In vivo: Male C57BL/6J mice were used to study the severe effects of FGF23. Male *Col4a3*^−/−^ mice (background 129 Sv/J), a model of human autosomal-recessive Alport syndrome and litter-matched WT mice were also used in this study	
Hindricks et al. (2014) [215]	FGF21	Human studies: Study cohort 1: Out of all 532 patients, those on PPARα- and PPARγ-agonists were excluded, and 499 patients stayed in the study that were categorized into CKD stages 1–5 according to the National Kidney Foundation—KDOQI guidelines Study cohort 2: 32 patients undergoing elective partial or total unilateral nephrectomy (model for acute kidney dysfunction) were enrolled	- In study cohort 1, circulating FGF21 was considerably dissimilar between CKD stages with maximum values detected in stage 5 when corrected for age, gender and body mass index - eGFR was a robust independent and negative predictor of FGF21 - In study cohort 2, FGF21 augmented appreciably post-surgery when matched to presurgical values - Moreover, relative changes in FGF21 levels were independently and positively associated with comparative changes in creatinine levels - Overall, FGF21 was elevated in both CKD and acute kidney disease

CHO: Chinese Hamster Ovary; CKD: Chronic Kidney Disease; ESRD: End-Stage Renal Disease; PTH: parathyroid hormone; eGFR: estimated Glomerular Filtration Rate; PHN: Passive Heymann Nephritis; WT: Wild-Type; UUO: Unilateral Ureteral Obstruction; PMN: Polymorphonuclear Neutrophils; PKA: Protein Kinase A; PLN: Proliferative Lupus Nephritis; NPLN: Non-Proliferative Lupus Nephritis; AIN: Interstitial Nephritis; CAN: Chronic Allograft Nephropathy; PPAR: Peroxisome Proliferator-Activated Receptor; KDOQI: Kidney Disease Outcomes Quality Initiative.

**Table 2:** Distinct roles of angiopoietin isoforms in diseases of the blood vessels, diabetes-associated complications and kidney.

Pathological Condition	Author and Year	Angiopoietin (Ang) Isoform	Main Findings and Conclusions
Vascular calcification	Chang et al. (2014) [295]	Ang-2	- The Ang-2 serum levels correlated independently with the severity of arterial stiffness in 416 CKD patients when measured by pulse wave velocity - Plasma levels of Ang-2 also augmented in mice subjected to 5/6 subtotal nephrectomy or UUO. Although Ang-2 was distinctly elevated in tubular epithelial cells of fibrotic kidneys, it was decreased in other tissues like aorta and lung, post 5/6 subtotal nephrectomy - Collagen and profibrotic genes in aortic vascular smooth muscle cells were up-regulated in mice with 5/6 subtotal nephrectomy and in mice generating human Ang-2 - Ang-2 induced expression of endothelial cytokines and adhesion molecules for monocytes, elevated aortic Ly6C (low) macrophages, and stimulated the expression of the profibrotic TGFβ1 in aortic endothelial cells and Ly6C (low) macrophages - Ang-2 blockade with recombinant protein L1-10 decreased the expression of monocyte chemokines, profibrotic cytokines, and collagen in the aorta of mice after 5/6 subtotal nephrectomy. Thus Ang-2 could be the missing link between kidney fibrosis and arterial rigidity
Diabetic retinopathy	(1) Joussen et al. (2002) [296]	Ang-1	- Intravitreal administration of Ang-1 to newly diabetic rats, stabilized retinal VEGF and intercellular adhesion molecule-1 mRNA and protein levels, resulting in reduced leukocyte adhesion, endothelial cell damage, and blood-retinal barrier collapse - Similarly, Adenovirus-Ang-1 when administered systemically to mice with established diabetes, repressed leukocyte adhesion and endothelial cell damage and blood-retinal barrier collapse - These alterations were accompanied by a decline in recognized facilitators of VEGF bioactivity and leukocyte adhesion, namely, retinal eNOS, nitric oxide, Akt (protein kinase B), and MAP kinase - Overall, these results reveal novel vascular and anti-inflammatory bioactivities for Ang-1 and identify its role in directly guarding the retinal vasculature in diabetes
	(2) Hammes et al. (2004) [297]	Ang-2	- The expression of Ang-2 and -1 in relation to the evolution of pericyte loss in diabetic rat retinae was studied, using quantitative retinal morphometry, and in retinae from mice with heterozygous Ang deficiency (Ang-2 LacZ knock-in mice). Recombinant Ang-2 was injected into eyes of nondiabetic rats, and pericyte numbers were quantitated in retinal capillaries - Ang-1 was present in the normal maturing retina and was upregulated 2.5-fold in diabetic retinae over 3 months of diabetes - In contrast, Ang-2 was consistently upregulated more than 30-fold in the retinae of diabetic rats, preceding the onset of pericyte loss - Heterozygous Ang-2 deficiency completely prevented diabetes-induced pericyte loss and reduced the number of acellular capillary segments - Injection of Ang-2 into the eyes of normal rats induced a dose-dependent pericyte loss. These data show that upregulation of Ang-2 plays a critical role in the loss of pericytes in the diabetic retina
	(3) Pfister (2010) [298]	Ang-2	- The effects of retinal overexpression of human Ang-2 (mOpsinhAng2 mouse) on vascular morphology in non-diabetic and streptozotocin-induced diabetic animals were investigated. Pericyte (PC) coverage and acellular capillary (AC) formation were quantitated in retinal digest preparations after 3 and 6 months of diabetes duration - The degree of retinopathy in non-diabetic mOpsinhAng2 mice at 3 months (−21% PC, +49% AC) was comparable to age-matched diabetic wild type mice - Diabetic mOpsinhAng2 mice exhibited significantly worse vascular pathology than wild type counterparts at 6 months. Quantitative PCR revealed that human Ang-2 mRNA was highly overexpressed in retinas of transgenic mice - Overexpression of Ang-2 in the retina enhances vascular pathology, indicating that Ang-2 plays an essential role in diabetic vasoregression via destabilization of pericytes
	(4) Rangasamy (2011) [299]	Ang-2	- Ang-2 mRNA and protein increased in the retinal tissues after 8 weeks of diabetes and in high-glucose-treated cells. Intravitreal injection of Ang-2 in rats produced a significant increase in retinal vascular permeability - Ang-2 increased human retinal endothelial cells monolayer permeability that was associated with a decrease in VE-cadherin and a change in monolayer morphology - High glucose and Ang-2 produced a significant increase in VE-cadherin phosphorylation - Increased Ang-2 alters VE-cadherin function, leading to increased vascular permeability. Thus, Ang-2 may play an important role in increased vasopermeability in diabetic retinopathy
	(5) Park (2014) [300]	Ang-2	- Pericyte loss occurred with an Ang-2 increase in the diabetic mouse retina and that the source of Ang-2 could be the endothelial cell. Ang-2 induced pericyte apoptosis via the p53 pathway under high glucose, whereas Ang-2 alone did not induce apoptosis - Integrin, not Tie2 receptor, was involved for Ang-2-induced pericyte apoptosis under high glucose as an Ang-2 receptor. High glucose changed the integrin expression pattern, which increased integrin α3 and β1 in the pericyte - Furthermore, Ang-2-induced pericyte apoptosis in vitro was effectively attenuated via p53 suppression by blocking integrin α3 and β1 - Although intravitreal injection of Ang-2 induced pericyte loss in C57BL/6J mice retina in vivo, intravitreal injection of anti-integrin α3 and β1 antibodies attenuated Ang-2-induced pericyte loss. In conclusion, Ang-2 induced pericyte apoptosis under high glucose via α3β1 integrin
	(6) Cahoon (2015) [301]	Ang-1	- In early diabetic retinopathy, adeno-associated virus serotype 2 encoding a more stable, soluble, and potent form of Ang-1 (AAV2.COMP-Ang-1) restored leukocyte-endothelial interaction, retinal oxygenation, vascular density, vascular marker expression, vessel permeability, retinal thickness, inner retinal cellularity, and retinal neurophysiological response to levels comparable with nondiabetic controls - In late diabetic retinopathy, AAV2.COMP-Ang-1 enhanced the therapeutic benefit of intravitreally delivered endothelial colony-forming cells by promoting their integration into the vasculature and thereby stemming further visual decline - AAV2.COMP-Ang-1 single-dose gene therapy can prevent neurovascular pathology, support vascular regeneration, and stabilize vision
	(7) Yun (2016) [302]	Ang-2	- Vascular leakage occurred with astrocyte loss in early diabetic mice (streptozotocin-induced diabetic retinopathy) retina as Ang-2 increased. The astrocyte loss and vascular leakage were inhibited by intravitreal injection of Ang-2-neutralizing antibody - In vitro, Ang-2 aggravated high glucose-induced astrocyte apoptosis via GSK-3β activation. Ang-2 directly bound to αvβ5 integrin, which was abundant in astrocyte, and the blockade of αvβ5 integrin, in vitro, effectively attenuated Ang-2-induced astrocyte apoptosis - In vivo, intravitreal injection of anti-αvβ5-integrin antibody inhibited astrocyte loss in early diabetic retinopathy. Taken together, Ang-2 induced astrocyte apoptosis under high glucose via αvβ5-integrin/GSK-3β/β-catenin pathway, and hence Ang-2/integrin signaling could be a potential therapeutic target to prevent the vascular leakage by astrocyte loss in early diabetic retinopathy
Diabetic nephropathy	(1) Lee (2007) [303]	Ang-1	- COMP-Ang-1 reduced albuminuria and decreased mesangial expansion, thickening of the glomerular basement membrane and podocyte foot process broadening and effacement - COMP-Ang-1 suppressed both renal expression of intercellular adhesion molecule-1 and monocyte chemoattractant protein-1 and monocyte/macrophage infiltration in diabetic db/db mice - COMP-Ang-1 also reduced renal tissue levels of TGFβ1, alpha-smooth muscle actin, fibronectin, as well as Smad 2/3 expression, but increased Smad 7 - In HUVECs grown in high glucose concentrations of glucose, recombinant COMP-Ang-1 protein decreased nuclear factor-kappaB (NF-kappaB) expression. COMP-Ang-1-mediated inhibition of increased NF-kappaB-DNA binding in nuclear extracts from HUVECs grown in high glucose was significantly blocked by soluble Tie2 receptor-Fc - In addition, COMP-Ang-1 significantly decreased fasting blood glucose level, epididymal fat weight to body weight ratio, and epididymal adipocyte size in diabetic db/db mice. After intraperitoneal glucose challenge, COMP-Ang-1 significantly lowered plasma glucose levels and thus, in conclusion, delayed the fibrotic changes in the kidney of diabetic db/db mice through its anti-inflammatory or metabolic effects
	(2) Davis (2007) [304]	Ang-2	- When the transgene was induced in mice with inducible podocyte-specific Ang-2 overexpression for up to 10 weeks, mice had significant increases in both albuminuria and glomerular endothelial apoptosis, with significant decreases of both vascular endothelial growth factor-A and nephrin proteins, critical for maintenance of glomerular endothelia and filtration barrier functional integrity, respectively - There was, however, no significant change of systemic BP, creatinine clearance, or markers of renal fibrosis, and podocytes appeared structurally intact. In kidneys of young animals in which Ang-2 had been upregulated during organogenesis, increased apoptosis occurred in just-formed glomeruli - In vitro, short-term exposure of isolated wild-type murine glomeruli to exogenous Ang-2 led to decreased levels of vascular endothelial growth factor-A protein. These novel results provide insight into molecular mechanisms underlying proteinuric disorders involving Ang-2
	(3) Dessapt-Baradez (2014) [305]	Ang-1	- Decreased Ang-1, VEGF-A upregulation, decreased soluble VEGFR-1, and increased VEGFR-2 phosphorylation (pVEGFR-2) was observed in streptozotocin-induced type 1 diabetic mice, accompanied by marked albuminuria, nephromegaly, hyperfiltration, glomerular ultrastructural alterations, and aberrant angiogenesis - Podocyte-specific inducible repletion of Ang-1 in diabetic mice caused a 70% reduction of albuminuria and prevented diabetes-induced glomerular endothelial cell proliferation; hyperfiltration and renal morphology were unchanged - Ang-1 repletion in diabetic mice increased Tie2 phosphorylation, elevated soluble VEGFR-1, and was paralleled by a decrease in pVEGFR-2 and increased endothelial nitric oxide synthase Serine (1177) phosphorylation - Diabetes-induced nephrin phosphorylation was also reduced in mice with Ang-1 repletion. Ang-1 therapy could be a renoprotective tool in diabetic nephropathy
	(4) Luo (2014) [306]	Ang-2	- Alprostadil treatment caused a significant decrease in the renal damage parameters in streptozotocin-induced diabetic nephropathy - Both Ang-2 and IL-18 were significantly increased in these mice and in glomerular endothelial cells cultured in high glucose; however, their expression was greatly reduced by alprostadil treatment - Ang-2 could also increase IL-18 expression in cultured endothelial cells under high glucose, and this response was partially blocked by Ang-2 siRNA - Ang-2 and IL-18 may be associated with the development and progression of diabetic nephropathy in mice
	(5) Khairoun (2015) [307]	Ang-1 + Ang-2	- An increase in the capillary tortuosity index in streptozotocin-induced + atherogenic diet (DM + ATH) pigs was reported as compared to the control groups. Kidney biopsies showed marked glomerular lesions consisting of mesangial expansion and podocyte lesions - A disturbed Ang-2/Ang-1 balance was observed in the cortex of the kidney, as evidenced by increased expression of Ang-2 in DM + ATH pigs as compared to control pigs - In the setting of diabetes mellitus, atherogenesis leads to the augmentation of mucosal capillary tortuosity, indicative of systemic microvascular damage, while, an imbalance in renal angiopoietins was correlated with the development of diabetic nephropathy
Chronic kidney disease (CKD)	(1) David et al. (2010) [308]	Ang-2	- 44 untreated non-smokers with varying stages of CKD 1–4 and 19 patients on dialysis (CKD stage 5) were recruited for measuring Ang-2 levels. Ang-2 measurements were also recorded in 15 healthy subjects prior to and 72 h after kidney donation - The median Ang-2 levels gradually augmented within the following groups: healthy controls: 0.77 ng/mL; CKD 1: 0.83 ng/mL; CKD 2: 0.93 ng/mL; CKD 3: 1.13 ng/mL; CKD 4: 1.75 ng/mL; and CKD 5: 4.87 ng/mL - Ang-2 was linked to the extent of CKD as supported by an inverse association with the GFR and positive association with homocysteine and phosphate - Also, Ang-2 positively correlated with the nitric oxide synthase inhibitor; asymmetric dimethylarginine levels and had inverse association with the mean alterations in GFR at 72 h post kidney donation
	(2) David et al. (2012) [309]	Ang-2	- In 128 CKD patients (43 CKD Stage 4, 85 CKD Stage 5 (57 hemodialysis, 28 peritoneal dialysis)), Ang-2 levels were considerably greater than in controls - Ang-2 was considerably greater in dialysis than in Stage 4 CKD patients and was associated with indicators of vascular disease (cholesterol, hsCRP, osteoprotegerin (OPG)) - Increased Ang-2 did not correlate with the extent of vascular calcification or with arterial stiffness - Cox-regression analysis concluded Ang-2 as an independent predictor of mortality in CKD patients
	(3) Chang et al. (2013) [310]	Ang-2	- 416 CKD patients were classified into stages 3 to 5 by urine albumin-creatinine ratio as normoalbuminuria (<30 mg/g), microalbuminuria (30–300 mg/g), or macroalbuminuria (>300 mg/g). Ang-2 and VEGF were increased, and soluble Tie2 levels in the plasma were reduced in the subgroups of albuminuria; with Ang-1 levels remaining unchanged - Linear regression analysis revealed a positive association between urine ACR and plasma Ang-2 only and not VEGF or soluble Tie2. Multivariate linear regression studies exhibited that plasma Ang-2 levels independently correlated with ACR and extremely sensitive C-reactive protein - In conclusion, plasma Ang-2 was related to albuminuria and markers of systemic microinflammation in CKD patients
	(4) Tsai et al. (2014) [311]	Ang-2	- In 621 patients with stages 3–5 CKD, 224 patients (36.1%) proceeded to begin dialysis and 165 (26.6%) reached doubling creatinine. 85 subjects (13.9%) had a quick decay in renal function. Ang-2 quartile was divided at 1494.1, 1948.8, and 2593.1 pg/mL - The linear mixed-effects model demonstrated a further swift decline in estimated glomerular filtration rate over time in patients with quartile 3 or more of Ang-2 than those with the lowest quartile of Ang-2 - Ang-2 could be an independent predictor of severe renal outcome in CKD
	(5) Bi et al. (2016) [312]	Ang-1	- Ang-1 appreciably reduced the angiotensin II-stimulated expression of the ER stress response proteins GRP78, GRP94, p-PERK, and CHOP, suggesting that Ang-1-facilitated cellular protection happens after the ER stress response - Tie2 inhibition using inhibitors and siRNA overturned these observations, inferring the prominence of Tie2 activation in preventing ER stress - The shielding effects of Ang-1 were attributed to the activation of ERK1/2 and p38 MAPK, which were significantly reduced when inhibited with specific inhibitors of these pathways (PD98059 and SB203580 respectively), along with moderate increase in the expression of chaperones involved in folding proteins - In conclusion, Ang-1 reduced ER stress-mediated cellular dysfunction and death via the Tie2 receptor/ERK1/2-p38 MAPK pathways in glomerular endothelial cells, which are principally associated with CKD

VEGF: Vascular Endothelial Growth Factors; HUVECs: Human Umbilical Vein Endothelial Cells; BP: blood Pressure; DM: Diabetes Mellitus; ATH: Atherogenic; OMP: Cartilage Oligomeric Matrix Protein; GFR: Glomerular Filtration Rate; ACR: Albumin-Creatinine Ratio; ER: Endoplasmic Reticulum.

**Table 3:** Distinct roles of angiopoietin isoforms in diseases of the bowel, lungs, and skin.

Pathological Condition	Author and Year	Angiopoietin (Ang) Isoform	Main Findings and Conclusions
IBD	(1) Koutroubakis et al. (2006) [313]	Ang-2	- In 160 IBD patients (79 UC and 81 CD) and in 80 corresponding healthy controls, median serum Ang-2 and Tie2 levels were notably greater in both the UC and CD patients in comparison to the healthy controls - The IBD patients detected early (diagnosis < 2 years) had considerably greater median serum Ang-2 levels but lower median serum Tie2 levels in comparison to patients with late IBD (diagnosis > 2 years) - The CD patients with dynamic disease showed appreciably greater levels of Ang-2 than in non-active disease patients. Interestingly, levels of both Ang-2 and Tie2 in the serum did not associate with laboratory markers such as ESR, CRP, white blood cell number, platelet count and albumin levels
	(2) Ganta et al. (2010) [314]	Ang-2	- Numerous main alterations were observed in the development of IBD in Ang-2(^−/−^) mice. Weight variations and disease activity differences were insignificant in WT and Ang-2(^−/−^) + DSS treated mice, while leukocyte intrusion, inflammation and blood and lymphatic vessel density was substantially diminished compared to WT + DSS mice - Gut capillary friability and water export appeared considerably earlier in Ang-2(^−/−^) + DSS mice vs. WT. Also, colon sizes were appreciably condensed in Ang-2(^−/−^) and gut histopathology was less impaired in Ang-2(^−/−^) in comparison to WT + DSS treated mice - The reduction in serum protein concentration in WT + DSS was less severe in Ang-2(^−/−^) + DSS, consequently PLE a characteristic trait of IBD was relieved by Ang-2(^−/−^) - In conclusion, in DSS colitis, Ang-2 facilitated inflammatory hemangiogenesis, lymphangiogenesis, and neutrophil intrusion to alleviate some clinical characteristics of IBD
	(3) Oikonomou et al. (2011) [315]	Ang-1 + Ang-2	- In 52 patients with UC, 59 with CD, and 55 healthy controls (HC), Ang-1 concentrations were considerably smaller in IBD patients compared to HC and were increased in smokers compared to non-smoker UC patients - IBD patients showed elevated Ang-2 levels compared to HC, whereas CD patients (disease only in colon) had notably lower Ang-2 levels when compared to other disease sites
	(4) Algaba et al. (2014) [316]	Ang-1	- In 37 patients with IBD treated with infliximab (16 with Crohn’s disease and 6 with ulcerative colitis) or adalimumab (15 with Crohn’s disease) and 40 healthy control subjects, Ang-1 levels diminished prior to each treatment dose in patients who achieved retardation of the disease - Elevated baseline VEGF levels anticipated for a dismal response to anti-TNF-alpha therapy, while elevated Ang-1 levels were linked with disease reduction - Serum VEGF and Ang-1 levels decreased post anti-TNF-alpha therapy and thus could be good predictors of response to therapy against IBD
	(5) Liu et al. (2015) [317]	Ang-1 + Ang-2	- Dysplasia and cancer were investigated in rats that received three rounds of 3.5% DSS with intraperitoneal pretreatment of DMH (CRC group). Colitis was investigated in rats that received three rounds of 3.5% DSS and intraperitoneal pretreatment with saline in UC group - CRC and UC groups exhibited the symptoms of serious colitis with diarrhea, rectal bleeding, wasting, and weight loss compared to controls - The mean length of the colon of was suggestively shorter in the CRC and UC group than in control rats. Only the CRC group had multiple tumors in the colorectal area (absent in UC and controls) - CRC and UC groups had strikingly augmented levels of Ang-1, Ang-2, Tie2, and VEGF protein in the colorectum compared to control group - In conclusion, aberrant expression of Ang-1, Ang-2, Tie2, and VEGF in UC-originated colorectal cancer may culminate in chronic colitis and pathologic angiogenesis
PH	(1) Zhao et al. (2003) [318]	Ang-1	- Ang-1 cDNA or null (pFLAG-CMV-1) vector was transfected into rat pulmonary artery smooth muscle cells. Fisher 344 rats were treated with monocrotaline (MCT) with or without transfer of 5 × 10(5) Ang-1 or null-transfected cells through the right jugular vein - 28 days post gene delivery, plasmid-derived Ang-1 mRNA was steadily and strongly expressed as assessed by RT-PCR in lungs from Ang-1 gene therapy group. Although Tie2 levels were noticeably reduced in rats treated with MCT, this effect was partly nullified by Ang-1 gene therapy - MCT-treated animals demonstrated 77% mortality by 28 days, while in pAng-1-treated animals, mortality was just 14% by 28 days. Moreover, measurement of the right ventricular systolic pressure and the right to left ventricular plus septal weight ratio was decreased in the Ang-1 group, as compared to MCT-treated group - Ang-1 gene transfer prevented the increased endothelial apoptosis and reduced endothelial NO synthase mRNA levels observed in the MCT-treated animals - Thus, cell-based gene therapy of Ang-1 enhanced survival and pulmonary hemodynamics in animal model of PH
	(2) Sullivan et al. (2003) [319]	Ang-1	- Constitutive Ang-1 expression (Adeno-Ang-1) in the lung of genetically engineered animals resulted in severe PH. Aberrant proliferation of smooth muscle cells (hyperplasia) causes diffuse medial thickening in small pulmonary vessels in these animals, a manifestation commonly observed in human PH - Ang-1/Tie2 signaling stimulated pulmonary arteriolar endothelial cells to manufacture and secrete serotonin (5-hydroxytryptamine), a strong smooth muscle mitogen. Also, elevated levels of serotonin were detected in human and rodent pulmonary hypertensive lung tissue as well - In conclusion, Ang-1/Tie2/serotonin paracrine pathway mediated pulmonary hypertensive vasculopathy, making them potential therapeutic targets to treat PH
	(3) Dewachter (2006) [320]	Ang-1	- PA-SMCs and P-ECs were obtained and grown from PH patients. Tie2 expression was 4-fold elevated in lungs and P-ECs from these patients compared to controls, accompanied with an equivalent escalation in phosphorylated lung Tie2 - However, Ang-1 and Ang-2 levels in lungs, P-ECs, and PA-SMCs were similar. Cultivation of PA-SMCs with medium collected from P-EC cultures provoked distinct proliferation. This effect was enhanced in P-ECs from PH patients rather than from control population - When P-ECs from either PH patients or control subjects were pre-incubated with Ang-1, they produced an additional increase in PA-SMC proliferation Fluoxetine, an inhibitor of the mitogenic activity of serotonin, decreased the proliferative effect of P-EC medium - Although Ang-1 improved the production of ET-1, serotonin, the amount of tryptophan hydroxylase-1 (the rate-limiting enzyme of serotonin synthesis) mRNA, preproET-1, and ET-1-converting enzyme when added to P-ECs from PH patients, platelet-derived growth factor-BB or epidermal growth factor levels were particularly unaffected - Ang-1/Tie2 signaling is involved in PH and contributes to PA-SMC hyperplasia through enhanced stimulation of endothelium-resulting growth factors synthesized by P-ECs
	(4) Kugathasan et al. (2009) [321]	Ang-1	- Right ventricular systolic pressure was moderately elevated in Tie2-deficient mice [Tie2(^+/−^)] when compared with WT littermate controls. The pressure was worsened when chronically stimulated with clinically significant PAH that triggered 5-HT or IL-6 - Although excess Ang-1 expression in transgenic mice had no adverse effect on pulmonary hemodynamics, it diminished the reaction to 5-HT. Incubation with 5-HT or IL-6 also reduced lung Ang-1 expression and subsequent Tie2 activation, thus promoting apoptosis in the Tie2(^+/−^) group lungs - Tie2 knockdown led to enhanced sensitivity to apoptosis after 5-HT treatment in cultured pulmonary artery endothelial cells - Z-VAD (pan-caspase inhibitor) treatment of Tie2-deficient mice, prohibited the pulmonary hypertensive reaction to 5-HT. Together these results decisively ascertain that the Ang-1-Tie2 pathway mediates the protective endothelial survival signaling in PH
	(5) Kümpers et al. (2010) [322]	Ang-2	- Plasma levels of Ang-1, Ang-2, soluble Tie2 (sTie2), and VEGF were increased in IPAH patients compared with controls. Among the angiogenic growth factors, Ang-2, but not Ang-1, sTie2, and VEGF were associated with cardiac index, PVR, and SvO(2) - Greater Ang-2 expression was an independent risk factor for mortality. 3 months after initiation of therapy, changes in Ang-2 levels paralleled with the variations in mean right atrial pressure, PVR and were inversely related to the variations in SvO(2) - Plexiform lesions from IPAH lung tissue showed increased expression of Ang-2 mRNA and protein. These findings suggest that Ang-2 may take part in the pathogenesis of IPAH, and circulating Ang-2 might serve as a potential diagnostic biomarker for determining disease gravity and treatment efficacy in IPAH patients
	(6) Kim D and Kim H (2014) [323]	Ang-1	- Circulating endostatin and Ang-1 in early life were linked to the development of PH in preterm infants with extreme BPD - The PH group regularly undertook more assertive respiratory management than the non-PH group, over 1 month after birth - The PH group demonstrated remarkably greater endostatin level and the ratio of endostatin to Ang-1 on day 7 of life when compared to the non-PH group or no/mild BPD groups - Additionally, the ratio of endostatin to Ang-1 on day 1 was considerably greater in the PH group in comparison to the no/mild BPD group - In conclusion, serum endostatin to Ang-1 ratio may represent compromised angiogenesis, which may play a role in the development of PH
Psoriasis	(1) Kuroda et al. (2001) [324]	Ang-1 + Ang-2	- Involved psoriasis skin demonstrated increased Ang-1, Ang-2, and Tie2 expression as compared to uninvolved psoriasis skin, healthy skin, and persistent spongiotic dermatitis skin. Extremely vascularized papillary dermis of involved psoriasis skin showed Ang-1 expression in the stromal cells, while Ang-2 was observed in endothelial cells around the multiplying epidermis that richly expressed VEGF - Aberrant expression of VEGF and FGF2 in involved psoriasis skin led to increased Ang-2 and Tie2 expression in dermal microvascular endothelial cell cultures - Overexpression of Ang-1, Ang-2, and Tie2 was related to the development of microvascular spread in psoriasis, suggesting that the Ang-Tie2 pathway may coordinate with VEGF and FGF2 together to foster neoangiogenesis in psoriasis - 5 psoriatic patients treated with PUVA (psoralen and ultraviolet A radiation) and two patients treated with tazarotene showed evident decrease in Ang-2 expression in skin lesions, indicating that modulation of Ang-2 may be crucial in regulating vascular proliferation in anti-psoriatic treatments
	(2) Markham et al. (2006) [325]	Ang-1 + Ang-2	- 16 patients with moderate to severe psoriasis and associated psoriatic arthritis (*n* = 13) were given infliximab infusions at baseline and at 2 and 6 weeks. The baseline levels of Ang-1/2, VEGF, Tie2, and TNF-alpha mRNA and protein were greater in pre-involved skin in comparison to uninvolved skin - Infliximab significantly reduced Ang-2, VEGF and Tie2 protein expression along with a reduction in Ang-1 and Tie2 mRNA expression. This was accompanied by a substantial decrease in the inflammatory infiltrate scores and CD31 expression, signifying inactivation of endothelial cell machinery - 12 weeks after treatment, there was a 93% mean decrease in the Psoriasis Area and Severity Index, and a noteworthy reduction in Disease Activity Score 28 and mean Health Assessment Questionnaire scores. These results decisively indicate that TNF-alpha is an important watchdog of the Ang/Tie2 pathway

IBD: Inflammatory Bowel Disease; CRC: Colorectal Cancer; UC: Ulcerative Colitis; CD: Crohn’s Disease; ESR: Erythrocyte Sedimentation Rate; CRP: C-reactive Protein; DSS: Dextran Sodium Sulfate; PLE: Protein losing Enteropathy; UC: Ulcerative Colitis; HC: Healthy Controls; DMH: 1,2-dimethylhydrazine; MCT: Monocrotaline; PH: Pulmonary Hypertension; RT-PCR: Reverse Transcriptase-Polymerase Chain Reaction; PA-SMCs: Pulmonary artery smooth muscle cells; P-ECs: Pulmonary Endothelial Cells; ET-1: Endothelin-1; 5-HT: Serotonin; IL-6: Interleukin-6; IPAH: Idiopathic Pulmonary Arterial Hypertension; PVR: Pulmonary Vascular Resistance; SvO(2): Mixed venous Oxygen saturation; BPD: Bronchopulmonary Dysplasia; CD31: Platelet Endothelial Cell Adhesion Molecule; TNF: Tumor Necrosis Factor.
